# Overview of Methods for the Direct Molar Mass Determination of Cellulose

**DOI:** 10.3390/molecules200610313

**Published:** 2015-06-04

**Authors:** Josua Timotheus Oberlerchner, Thomas Rosenau, Antje Potthast

**Affiliations:** Christian-Doppler Laboratory “Advanced Cellulose Chemistry and Analytics”, Division of Chemistry of Renewable Resources, Department of Chemistry, University of Natural Resources and Life Sciences Vienna, Muthgasse 18, A-1190 Vienna, Austria; E-Mails: josua.oberlerchner@boku.ac.at (J.T.O.); thomas.rosenau@boku.ac.at (T.R.)

**Keywords:** cellulose, molar mass distribution, molar mass average, viscometry, end group analysis, osmometry, ultracentrifuge, size exclusion chromatography, light scattering, cello-oligomers

## Abstract

The purpose of this article is to provide the reader with an overview of the methods used to determine the molecular weights of cellulose. Methods that employ direct dissolution of the cellulose polymer are described; hence methods for investigating the molecular weight of cellulose in derivatized states, such as ethers or esters, only form a minor part of this review. Many of the methods described are primarily of historical interest since they have no use in modern cellulose chemistry. However, older methods, such as osmometry or ultracentrifuge experiments, were the first analytical methods used in polymer chemistry and continue to serve as sources of fundamental information (such as the cellulose structure in solution). The first part of the paper reviews methods, either absolute or relative, for the estimation of average molecular weights. Regardless of an absolute or relative approach, the outcome is a molecular weight average (MWA). In the final section, coupling methods are described. The primary benefit of performing a pre-separation step on the molecules is the discovery of the molecular weight distribution (MWD). Here, size exclusion chromatography (SEC) is unquestionably the most powerful and most commonly-applied method in modern laboratories and industrial settings.

## 1. Introduction

### 1.1. Something about Cellulose

Cellulose is a commercially important biopolymer [1]. The term *bio* refers to plants, algae, bacteria, and animals; basically, anything that lives and is thus (at least in principle) a renewable resource. The properties of cellulose can vary significantly depending on the origin, the isolation process and/or the treatment. In this context the ability to determine cellulose’s molecular weight and its chemical uniformity is crucial. After nearly 200 years of polymer chemistry, the determination of polymer molecular weights (averages and distributions) remains a challenge. When Hermann Staudinger first described the molecular structure of macromolecules [2], there was considerable doubt concerning his hypothesis [3,4]. Today, it is generally accepted that polymers consist of repeating units that are bound together covalently. As a consequence, most polymers do not have individual molecular weights in the same way that glucose, for example, always has a molecular mass of 180 g∙mol^−1^. A polymer consists of molecules with different chain lengths (proteins are not included in this characterization), so their molecular weights are always average values. Averages offer an incomplete summarized description of the molecules molar mass. Initial attempts to determine the molecular weight of polymers were performed with natural polymers. Staudinger investigated cellulose and rubber by viscometry [5]. The appearance and ever-increasing role of synthetic polymers in daily life has led to a greater scope of analytical methods for polymer characterization.

In cellulose research, the contemporary method of choice for determining molecular weight is size exclusion chromatography (SEC) coupled with a light scattering detector and a refractive index (RI) detector. In industrial settings, viscometry (without separation) still plays a major role since it is quick and, at least in theory, does not require much expertise. Other methods can provide complementary information, however. In fact, a fundamental knowledge of different methods for determining molecular weight can be beneficial in understanding the cellulose molecule. Thus, the historical background of cellulose research should be considered, at least to a certain degree.

### 1.2. How to Define Polymer Molecules—Average Values

The number of monomer units in cellulose and for polymers in general is given as the degree of polymerization (DP). Methods that do not fractionate the sample can only provide an average of the molecular weight for all molecules. There are several forms of average values. The most prominent is likely the weight-average molecular weight *M_w_*, which can be directly obtained by light scattering measurements. The number average *M_n_* is important for calculating end groups or the dispersity *Đ.* Cellulose materials have DPs that vary depending on the source, production process, and treatment [1]. DP values range from 100–3000 for commercial celluloses to 20,000 for cotton fiber secondary walls to 44,000 for *Valonia*, which is a species of algae. Native cellulose generally has higher DP values than regenerated cellulose or cellulose processed by pulping. Several different *M_W_* averages can be defined (Equations (1)–(3)):

(1) $$Number\;average\;M_{n}=\frac{\sum^{\text{​}}n_{i}M_{i}}{\sum^{\text{​}}n_{i}}$$

(2) $$Weight\;average\;M_{w}=\frac{\sum^{\text{​}}n_{i}M_{i}^{2}}{\sum^{\text{​}}n_{i}M_{i}}$$

(3) $$Z-average\;M_{z}=\frac{\sum^{\text{​}}n_{i}M_{i}^{3}}{\sum^{\text{​}}n_{i}M_{i}^{2}}$$

where *n_i_* is the number of molecules with molar mass *M_i_*.

The dispersity is given by the ratio of the mass average molar mass to the number average molar mas (Equation (4)):

(4) $${Đ}_{M}=\frac{M_{w}}{\;M_{n}}$$

The *Đ_M_* is a general measure of the heterogeneity with regard to chain length. A *Đ_M_* of 1.000 refers to a single molecular mass, a *Đ_M_* close to 1 refers to a very narrow distribution. After the analysis that supplies the distribution of cellulose molecular weights, it is common to provide an average DP along with the *Đ_M_*.

**Figure 1:**
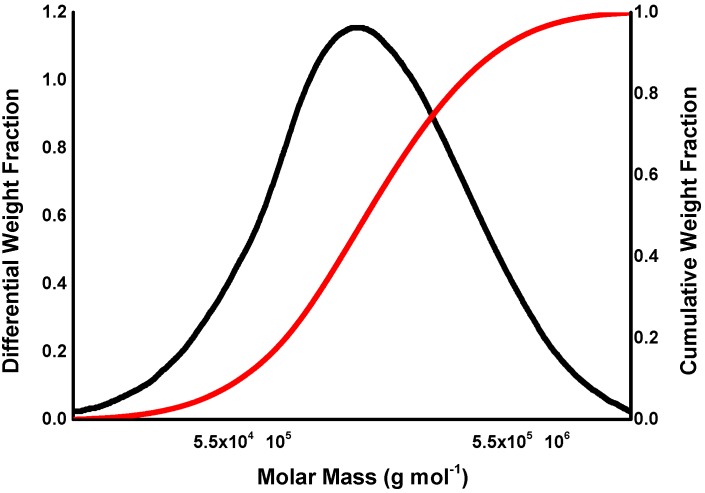
Differential (**black**) and cumulative (**red**) plots of MW.

Molecular weight distribution can also be described by distribution curves (graphically) or by distribution functions (mathematically). It is a common practice to express the molecular weight axis in a logarithmic scale. In cellulose analysis, the function *F_w_*(*log M*) is typically used for interpretation. The cumulative molecular weight is used less frequently but works well for comparisons (integral, Figure 1).

For non-uniform samples, the following relation holds true:

(5) $$M_{n}<M_{w}<M_{z}$$

For a uniform sample, all these values are the same. The impact of the higher molecular weights on the average increases along with the exponent in the numerator (Equations (1)–(3)). The relevance of any of the averages for a property depends on the extent to which the average in question takes into account the segment of the molar mass distribution that mainly influences the property. For example, the tensile strength is normally influenced by large molecules; therefore, the M_w_ relates molecular weight and property. For stoichiometric calculations or kinetic experiments, the number average is needed. M_n_ can be determined by using the colligative properties of a polymer solution, e.g., by using osmometry, by the end group method, or by light scattering after separation (e.g., SEC). The z-average can be obtained by ultracentrifuge experiments (see Chapter 2.3) or by light scattering techniques after separation. At times, M_z_ is used to describe flex life and stiffness, which are governed by the longest chains in the MWD. However, in cellulose analysis and chemistry, M_z_ has a low practical importance.

The viscosity average *M_v_* (see Section 2.4) and *M_w_* are often close to each other, hence viscometry is said to yield *M_w_* values; however, this is a common misconception. *M_w_* and *M_v_* can differ considerably, so viscosity measurements deliver only relative *M_v_* values.

### 1.3. An Important Requirement—Dissolving Cellulose 

A very important and major requirement for all the methods described is the dissolution of the polymer in a given solvent. For many polymers, dissolution can be a challenge. Since cellulose has a dense, partially crystalline structure and numerous intra- and intermolecular hydrogen bonds, it is virtually insoluble in most common solvents [6]. The goal is to bring the polymer molecules into a solution without degradation, aggregation, or association, which can only be achieved in very dilute solutions. Very dilute here means that a single molecule has more space than its own size in the solvent. A detailed description of the dissolution process for cellulose is not part of this review but can be found in several sources [7,8,9]. However, for molecular weight determination, only a few solvents are of interest and for the sake of clarity, they will be briefly described here (for more details, see the article “Solvents applied in the field of cellulose chemistry”) [10]. The first solvent for cellulose was discovered by the Swiss chemist Schweizer, who applied copper hydroxide-ammonia (cuoxam, called Schweizer’s reagent) for the dissolution of cellulose [11,12,13]. Schweizer’s reagent is still used in modern cellulose chemistry. Copper ethylenediamine (cuen or CED) is used to determine *M_v_* in standard industrial methods [14,15,16]. Cuoxam and cuen can directly dissolve cellulose in a relatively short time, but their drawback is the instability of cellulose in these solvents when the cellulose is oxidized (even if only to a very low extent), which leads to degradation by a beta-elimination mechanism.

*N*,*N*-Dimethylacetamide/lithium chloride (DMAc/LiCl) is the solvent most frequently used for analyzing non-derivatized cellulose in molecular weight distribution studies. This system shows enormous potential in cellulose chemistry, with uses ranging from analytics to the preparation of a wide variety of derivatives [10]. The dissolution process of cellulose in DMAc/LiCl was first described by McCormick in 1985 [17]. One year later, this solvent system was first used with SEC on cellulose [18]. Strlic reported the zero order random degradation constant of cellulose in 0.5% DMAc/LiCl with k = 6.9 × 10^−8^ mol·mol^−1^·monomer day^−1^, which means a degree of the molecular weight (*M_n_*) of 47 g·mol^−1^ per day. However, today, cellulose solutions in DMAc/LiCl at room temperature or below are considered stable. When using DMAc/LiCl without heating the sample, it can be assumed that this is a non-degrading solvent system [19,20,21].

Although Gränacher introduced *N*-methylmorpholine-*N*-oxide monohydrate (NMMO) as a cellulose solvent in 1939, NMMO was first used commercially in the 1980s [22]. NMMO is a very potent cellulose solvent; however, it only dissolves cellulose at elevated temperatures. Thus, NMMO has a more practical use in cellulose processing than in cellulose analytics, but it can also be used for viscometry. Eckelt *et al*. reported a relationship between data of cellulose dissolved in NMMO and cuen by means of viscometry [23].

The use of organic solvents for cellulose after derivatization is another way to dissolve cellulose molecules into a solution. However, it is not the aim of this article to review derivatization procedures for cellulose, as such methods can be found elsewhere (e.g., Klemm *et al*.) [24,25,26]. The derivatization of cellulose does have some drawbacks, such as the loss of low molecular weight material, the degradation of high molecular weight material, or the need for removal of lignin. In order to obtain accurate molecular weights, a direct dissolution is preferred, and it has been argued that a direct solution of the polymer provides a better chance of obtaining true molecular weights. Even in the early days of cellulose molecular weight determinations, Staudinger recognized the rapid degradation of cellulose in cuen solution and proposed a higher molecular weight than what he actually found [27]. A comprehensive discussion on this topic and a comparison of direct dissolution and derivatization techniques prior to SEC-MALLS is given in Potthast *et al.* [28].

**Figure 2:**
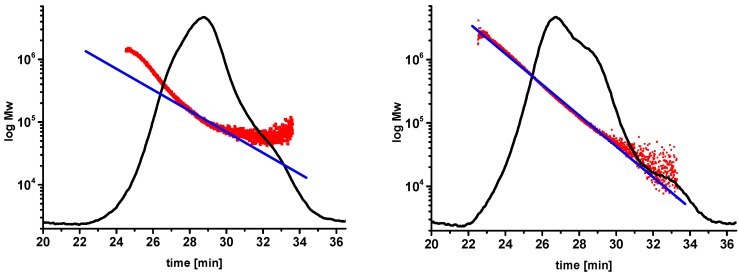
SEC-MALLS chromatograms of the same sample dissolved individually. The chromatogram on the left shows a poor state of dissolution; the chromatogram on the right depicts a satisfactory state of dissolution of the polymer. The red dots represent a light scattering signal; the blue lines represent a fitted (linearized) light scattering signal [34].

Whatever solvent system is used for the dissolution of cellulose, the aim is to get the polymer molecules into a molecular dispersion system without any degradation. A molecular dispersion can be seen as a true solution of a solute phase, represented by single cellulose molecules in a solvent. The molecules in the dispersed phase are separated from each other and homogenously distributed throughout the solvent. In the case of cellulose, as with other polysaccharides, different super- and supramolecular structures of the molecules in solution can occur; e.g., smaller and larger aggregates, single molecules, or fringed micelles [17,29]. Since light scattering is very sensitive to aggregates, it can be used to identify them. For SEC multi angle laser light scattering (SEC-MALLS) measurements, the light scattering signals together with the RI response yield information on the state of dissolution (Figure 2). In the case presented in Figure 2, a set of four linear columns has been used to measure the same independently prepared cellulose sample. The example on the left side shows a strong deviation from the expected linear relationship between retention time and molar mass. There can be numerous reasons for this, but in the case of cellulose, this effect appears to be mainly due to an insufficient state of dissolution. As a consequence, the MWD shows a clearly lower resolution over the MWD as a whole and in the hemicellulose region in particular, which appears below 32 min. In their work, Yamamoto *et al*. used the 90° signal from MALLS detection to check holocellulose (cellulose and hemicelluloses without lignin) solutions for aggregates [30]. Röder and Potthast investigated aggregate formation for cellulose solutions in DMAc/LiCl with varying amounts of water in the solution [21,31]. For chitosan, a linear biopolymer with glucosamine as the repeating unit, static light scattering was used successfully for the analysis of aggregates [32,33].

A common test to determine whether the polymer occurs as a molecular dispersed solution is to inject the same sample at different concentrations into a SEC-MALLS system. The MWD obtained should be independent of the mass injected. If the MWD changes significantly formation of aggregates can be assumed due to too high concentrations [35]. Röder *et al*. point out that the dilution error in static light scattering (SLS) can be quite large [36]. In addition, a non-linear angular dependence in the Guinier-Zimm Plot suggests the formation of aggregates. Röder *et al*. investigated the solution behavior of cellulose in technologically relevant solvents by means of static light scattering, dynamic light scattering (DLS), small-angle X-ray scattering (SAXS), and depolarized dynamic light scattering (DDLS) [37]. They showed that cellulose molecules are only molecularly dispersed in DMAC/LiCl 0.9% [38]. In industrially relevant solvent systems, cellulose chains often build up a loose network with gel particles (e.g., xanthogenate in the viscose process) or an entanglement network with highly swollen aggregates (NMMO, Lyocell process). This phenomenon depends to a great extent on the solute concentration. Offline DLS measurements are superior for the analysis of aggregation, as DLS can also capture aggregates quantitatively. In depolarized dynamic light scattering, even the structure of the fringed micelle can be investigated because the compact and anisotropic cores of the micelles are captured.

Cellulose can also be dissolved in aqueous (complex-forming) solvents or in organic solvents after derivatization (as a cellulose ester, e.g., cellulose acetate or cellulose nitrate; or as an ether, e.g., methyl cellulose). Today, cellulose tricarbanilates are employed by some groups because they allow the use of common organic solvents for SEC such as THF and the use of UV detection instead of RI, which offers a higher sensitivity. Care must be taken not to degrade the sample and not to discriminate the MWD during derivatization and workup [28]. Cellulose acetates (2.5 or 3 acetates) are not an option for SEC, as they are often not soluble without aggregation [39,40,41]. All solvents share the same main-dissolution mechanism, *i.e.*, the disruption of hydrogen bonds.

## 2. How to Measure Molecular Weights

### 2.1. End Group Method 

The molecular weight of polymers which have an end group amenable to analysis could be principally analyzed by an end-group assay [42]. The biggest drawback of this method is the decrease in sensitivity with an increased chain length. The first measurements with this method were performed by Staudinger and Eder and gave determination limits of about 15,000 g∙mol^−1^ [43]. Weber and Husemann treated cellulose with an alkaline copper sulfate solution to oxidize aldehydes to aldonic acids. Afterwards, they determined the so-called “*Monosezahl*” (number of glucose units per carboxyl group). With this method, they were able to determine molecular weights up to 200,000 g∙mol^−1^ [44,45]. However, due to processing, cellulose usually already contains oxidized end groups and additional carbonyl groups. Hence, a direct relationship between carboxyl groups (aldonic acid) and molecular weight determined with a high rate of accuracy.

### 2.2. Osmotic Pressure

Osmometry is among the methods for determining molecular mass which rely on colligative properties (from the Latin word *colligere* for “collect”), meaning that only the number of dissolved molecules influences the properties of a solution [46]. In addition, the osmotic pressure, boiling point elevation, vapor pressure reduction, and the freezing point depression are based on colligative properties. Out of the four colligative methods, only membrane osmometry (MO) is of interest for cellulosic samples. However, vapor pressure osmometry (VPO) is superior for analyzing samples with *M_n_* < 20,000 g∙mol^−1^. This is because the diffusion of low molecular weight molecules through the membrane limits the utility of membrane osmometry for this especially low molecular weight region. Only a few publications address the use of VPO experiments on directly-dissolved cellulose and these will be discussed at the end of this chapter.

Since the osmotic pressure of a solution depends on molecular weight and concentration *c*, the following equation can be used to determine the number average of the molecular weight:

(6) $$\frac{П}{c}=RT\left(\frac{1}{M_{n}}+A_{2}c+A_{3}c^{2}+\ldots \right)$$

where *П* is the osmotic pressure, *c* is the solute concentration, *R* is the ideal gas constant, *T* the temperature, *M_n_* is the number average, and *A*_2_ and *A*_3_ are the second and third virial coefficients. The most common method of osmometry is membrane osmometry. In membrane osmometry, the osmotic pressure is measured directly using a semi-permeable membrane [42]. In experiments, the osmotic pressure must be measured at several different concentrations. By extrapolating the *П*/*c* versus *c* plot to zero, the intercept gives the molecular weight, whereas slope yields *A*_2_. Note, that *A*_2_ is an empirical constant for a given solute/solvent system and it depends on the temperature. It represents the interaction of a single molecule with the solvent. For the fundamental theory of osmometry, see Hunt [47].

Using osmometry for cellulose molecular weight determination creates a few problems. First, the osmotic pressure is inversely proportional to molecular weight, so molecules with a high molecular weight contribute very little. Therefore, the sample must be free of low molecular compounds when applying osmometry. This is especially true for salts and, therefore, for aqueous cellulose solutions. This is the main reason that osmometry is ordinarily used with cellulose derivatives in organic solvents [48,49]. In most cases, cellulose-based membranes such as cellophane or bacterial cellulose are used for membrane osmometry [50]. However, these membranes are not completely resistant against solvents used for cellulose. Hence, the usual cellophane membrane gels which are used in an osmometer would dissolve in, for example, cuen solutions. Even poly(vinylbutyral) (PVB), which is often employed as a membrane, will likely dissolve in DMAc/LiCl [51].

Immergut *et al*. developed two membranes, Kel-F and leached polyvinyl butyral, that are stable against copper ethylenediamine solutions [52]. Working with those membranes, they measured the molecular weights of two cellulose samples directly. Since cellulose in cuen behaves as a polyelectrolyte, they faced another problem beyond membrane instability. However, by proper conditioning of the membranes, the polyelectrolyte character of the solution can be compensated and allow for a valid measurement. Immergut’s paper is one of the few dealing with the direct osmometry of pure cellulose. This method continues to play a minor role today, even for derivatized cellulose.

It is worth noting that osmometry was originally used to determine the relationship between intrinsic viscosity and molecular weight [53]. It has been shown that under mild conditions, the degradation during cellulose nitration is negligible. In his pioneering work, Staudinger used the osmometry of cellulose nitrates in acetone to determine the constant *K_cm_* of the Staudinger Equation (7):

(7) $$\log{\text{η}}_{r}=K_{cm}*M*c$$

where η*_r_* is the relative viscosity (see Chapter 2.4), *M* is the molecular weight, and *K_cm_* is the molecular weight-concentration constant. The Staudinger equation was later the basis for the Mark-Houwink equation (see Chapter 2.4).

Using VPO instruments with increased sensitivity, Kamide *et al*. detected an upper limit of 1 × 10^5^ g∙mol^−1^ [54]. Compared with the results for cellulose acetate obtained by MO and SEC, VPO values differed by only a small percentage [55].

### 2.3. Ultracentrifugation

When polymer chemistry was in its early stages in the 1920s, analytical ultracentrifugation experiments played an important role. Svedberg introduced two analytical ultracentrifugation methods: the sedimentation velocity method and the sedimentation equilibrium method [56]. The sedimentation velocity method (performed at, for example, 70,000 RPM) provides information on the physical homogeneity of a sample, its conformation, interaction or co-sedimentation, and flexibility information. The sedimentation equilibrium, which is carried out at lower rotor speeds such as 15,000 RPM, yields information on absolute weight averages (*M_w_*, and *M_z_*) and molecular weight distributions. Ultracentrifugation is also capable of measuring the molecular charge in polysaccharides. Diffusion parameters can also be obtained by ultracentrifugation experiments; however, strictly speaking, diffusion has nothing to do with ultracentrifugation, though it is very closely connected to the theoretical and practical background. See Vollmert [57] for a detailed theoretical discussion.

The first studies of cellulose in which ultracentrifugation was used were performed by Stamm between 1926 and 1930 [58,59,60]. Stamm investigated cellulose dissolved in cuprammonium (tetraamine copper(II) sulfate) and cellulose xanthogenate in diluted alkali solutions. He and his coworkers investigated the state of dispersion of cellulose in cuprammonium solutions. They found diffusion coefficients of cellulose and also claimed that cellulose was uniform with a molecular weight of 55,000 ± 7000 g∙mol^−1^. However, they realized that under an oxygen atmosphere, the cellulose is degraded. A typical equilibrium run time was 190 h; therefore, severe degradation of the molecule during the measurements can be assumed.

Gralén and Svedberg measured cellulose in cuprammonium solutions under a nitrogen atmosphere [61]. They calculated molecular weights for native cotton with 1000 kg∙mol^−1^ (DP 6200) and for Ramie with 1840 kg∙mol^−1^, which can be considered very close to values measured today using other techniques.

Jullander published the first review of studies on cellulose and its derivatives performed using ultracentrifugation experiments [62]. He concluded that it was possible to calculate weight averages from diffusion experiments and that number, weight, or z-averages can be obtained from sedimentation runs.

Marx and Meyerhoff calculated the molecular weight of cellulose based on a calibration done with ultracentrifugation experiments [63,64]. They found two molecular weight averages (maxima) for cotton and flax cellulose between DPs of approximately 5500 and 11,000, while for spruce they reported an average DP of 8000. In their conclusion, they noted the similarity of cellulose collected from different origins but did not give an explanation of bimodal distribution curves. The major limitation of ultracentrifugation experiments is the analysis time needed; the time to reach equilibrium can be up to several days or even weeks. In the area of synthetic polymers, ultracentrifugation experiments have been nearly completely replaced by other techniques. For biopolymers, especially cellulose, ultracentrifugation still plays a minor role in analytics. Two recent review articles published by Harding *et al*. [65,66] discuss the possibilities of ultracentrifugation experiments in polysaccharide analysis today; especially in combination with SEC and MALLS, it can provide complementary information such as the heterogeneity of materials. Like SEC-MALLS, the sedimentation equilibrium method provides a molecular weight distribution, although there is no column or separation device and therefore no limitations concerning the column’s medium inertness or available pore sizes.

Today, the ultracentrifuge is used to obtain fundamental biophysical information about solutions of cellulose rather than to determine molecular weight. In his perspective papers, however, Harding demonstrated that sedimentation equilibrium is a powerful and valuable independent check on the results generated with other methods such as SEC-MALLS experiments.

### 2.4. The Viscosity of Cellulose Solutions

Polymers are, in general, less soluble than their corresponding monomers. Dissolving polymers in solvents leads to an increase in viscosity; the longer the average chain length of the polymer molecules, the more the viscosity changes (known as positive viscosity). The viscosity of a solution can be easily measured; therefore, viscosity measurements are widely used in determining the average DP of cellulose.

In 1930, Hermann Staudinger was the first to recognize an empirical relationship between the relative magnitude of the increase in viscosity and the molecular weight of the polymer [27]. The simplest method for determining the viscosity of a polymer solution is by capillary viscometry, using the Ubbelohde U-tube viscometer. Here, the flow time *t* of the polymer solution and of pure solvent *t*_0_ are recorded. The ratio of the flow time of a polymer solution *t* to that of pure solvent is equal to the ratio of their viscosities (η/η_0_) if their densities are equal. This is only feasible for dilute solutions (in which density differences level out). Because unity is the lower limit of the relative viscosity η*_r_*, the specific viscosity η*_sp_* is more useful, as it depicts the relative increase brought about by the dissolved polymer.

Relating the specific viscosity to concentration gives the reduced viscosity. The intrinsic viscosity is expressed as the limit of the reduced viscosity at zero concentrations. The inherent viscosity is given as the natural logarithm of the relative viscosity divided by the concentration. At concentration zero the inherent viscosity becomes equal to the intrinsic viscosity. Thus, either the extrapolation of the reduced viscosity or the inherent viscosity gives the intrinsic viscosity:

(8) $$\text{Relative}\;\text{Viscosity}:\;{\text{η}}_{r}=\frac{\text{η}}{{\text{η}}_{0}}$$

(9) $$\text{Specific}\;\text{Viscosity}:\;{\text{η}}_{sp}=\frac{\text{η}-{\text{η}}_{0}}{{\text{η}}_{0}}={\text{η}}_{r}-1$$

(10) $$\text{Reduced}\;\text{Viscosity}:{\text{η}}_{red}=\frac{{\text{η}}_{sp}}{c}$$

(11) $$\text{Intrinsic}\;\text{Viscosity}:\left[\text{η}\right]=\;\underset{c\to 0}{\lim}\frac{{\text{η}}_{red}}{c}$$

(12) $$\text{Inherent}\;\text{Viscosity}:\;{\text{η}}_{inh}=\frac{\text{ln}\;{\text{η}}_{r}}{c}$$

For a given polymer-solvent pair the intrinsic viscosity is a unique function of molecular mass. The Mark-Houwink (or Kuhn-Mark-Houwink-Sakurada) equation then relates the molecular weight of the polymer plus solvent at a specified temperature to the intrinsic viscosity [4]:

(13) $$\left[\text{η}\right]=K_{v}M^{v}$$

*K_v_* (or sometimes *K_M_*) and *v* (sometimes notated *a*) must be established by calibrating with polymers of known molecular weights. Once this has been performed, only [η] will give the molecular weight for an unknown molecule, which is normally done by plotting log [η] against log *M* and subsequent interpolation.

For a theta solvent, *v* = 0.5, and as the solvent becomes thermodynamically better, *v* increases. For most practical systems, these values can be found in data handbooks. The extrapolation factors for cellulose to zero concentration are also listed. Under these assumptions, it is possible to calculate the intrinsic viscosity by measuring only one concentration of the sample. Molecular weights derived via the Mark-Houwink equation and viscometry yield the viscosity average molar mass [*M_v_*], which is given by:

(14) $$M_{v}={\left[\frac{\sum^{\text{​}}n_{i}M_{i}^{1+v}}{\sum^{\text{​}}n_{i}M_{i}}\right]}^{1/v}$$

where *n_i_* is the number of molecules of molar mass *M_i_* and the exponent *v* is the exponent of the Mark-Houwink equation. It should be noted that the viscosity average is not an absolute average and depends on solvent and/or temperature; therefore, viscosity measurements yield relative values. *M_v_* is not a fixed quantity—it depends on *v*. If *v* becomes unity, viscosity and weight average are equal; *M_v_* lies therefore, in between *M_n_* and *M_w_* in magnitude but will be usually closer to *M_w_*.

Viscometry is the leading method for determining average molecular weight in industrial applications. As a standalone method, it delivers *M_v_* values, typically by using an Ubbelohde capillary viscometer. In industrial applications, the measurement of [η] provides a quick and easy route to the molecular weight. For viscosity measurements, a minimum requirement is the knowledge of the dependence of [η] *on* (*M*).

The solvent most commonly used for viscometry measurements is the complex-forming solvent cuen, largely because of its rapidity and simplicity in dissolving cellulose. One drawback of cuen lies in the cuen solution’s high alkalinity, which may induce degradation reactions at oxidized functionalities. Cellulose solutions in cuen are not very stable, and the solubility for cellulose with high molecular weight (DP > 5000) is relatively poor.

Viscometry was actually one of the first methods used to determine polymer molecular weights. It was Hermann Staudinger who applied viscometry to cellulose analytics in the 1930s. Staudinger described the *K_m_* value (for the Staudinger equation) of cellulose in cuen with 1 × 10^−4^; in 1938 he published the *K_m_* values for cellulose in Schweizer’s Reagent as 5 × 10^−4^ [67].

It is important to remember that *K_v_* and *v* are empirical constants. There are several standard methods for estimating the degree of polymerization of a cellulose sample from its intrinsic viscosity in cuen solution. In process control practices in industrial environments, the intrinsic viscosity is often estimated from a single viscosity measurement. There are several published warnings when using such standard methods. Evans *et al*. showed that the Mark-Houwink-Sakurada (MHS) equations used in the SCAN C15:62 standards are not entirely correct [68]. The relationship between intrinsic viscosity and the DP (or the molecular weight) is very often derived from osmometric measurements of cellulose trinitrate samples in acetone. Such measurements date back to very early experiments and should be evaluated with modern methods. It is commonly assumed that the molecular weight average determined by viscosity is very close to *M_w_* and that *M_v_* therefore is an approximation of *M_w_*. In fact, *M_v_* can often be very far from *M_w_*, depending on the MHS parameters used and on the molecular weight distribution of the sample. Only if the sample is very narrowly distributed or if uniform standards are used can *M_v_* be used as an approximation of *M_w_*. MHS parameters found in the literature can be excessively variable, with *K_v_* values ranging from 0.42 to 1.87 and *v* values ranging from 0.771 to 0.905 [69].

In spite of these factors, viscometry remains a primary method for obtaining cellulose molecular weight averages because it does not require calibration. It is also a feasible method for monitoring cellulose degradation during process steps, which results in relative data. This is mainly because it does not require advanced equipment, the method is relatively simple, and it is specifically recommended by industrial standards. It is essential to remember that the parameters for the Mark-Houwink equations are specified for cellulose and that it is problematic to use such parameters for samples with, for example, high lignin or hemicellulose content.

The equations also depend on the expected DP range; a DP larger than 950 requires a different equation than the equation used for lower DPs. According to the current knowledge, there is no equation available for cellulose with a large oligomeric portion, so the cuen-DP values in such cases may be prone to error. It is standard practice to use linear fits for data when drawing a *log* [*n*]*-log M_w_* plot. In general, there is no justification for the linearity, and it is questionable whether such fits are applicable for cellooligomers or samples containing oligomers in such high amounts [68].

Thus far, most Mark-Houwink equations for cellulose were established for cellulose in cuen or cadoxen solutions [68,69,70] because these solvents demonstrate beneficial properties for the rapid dissolution of cellulose. However, the instability of cellulose in this solvent must be noted, as should the limited dissolving power for very large molecules. Kasaai *et al*. [71] analyzed the intrinsic viscosity of cellulose in different solvents and reported the following viscosity order:

(15) $${\left[\text{η}\right]}_{/LiCl\;}>\;{\left[\text{η}\right]}_{NH3/NH4SCN\;}\geq {\left[\text{η}\right]}_{FeTNa\;}>{\left[\text{η}\right]}_{cuen}>{\left[\text{η}\right]}_{cadoxen}>{\left[\text{η}\right]}_{cuoxam}$$

An analysis of the stability indicates that degradation was the lowest in DMAc/LiCl/ and NH_3_/NH_4_SCN, moderate in cadoxen and ferric chloride/sodium tartrate/sodium hydroxide (FeTNa), and highest in cuen and cuoxam. The viscosity of cellulose in NMMO, a very important solvent within the technology sector, was first measured by Eckelt *et al*., who reported the following equation [23]:

(16) $$log\;(\frac{\left[\text{η}\right]}{\text{ml}*g^{-1}})=\;-1.465\;+\;0.735\;log\;M$$

The Kuhn Mark-Houwink plot for cellulose in NMMO∙H_2_O shows a non-linear behavior of the fit. After reaching a critical value, the straight line deviates. Thus far, this study is the only paper in which the cellulose viscosity in NMMO solutions is addressed. For analytical purposes, this solvent system has no practical importance because of the elevated temperature it requires.

### 2.5. Light Scattering Methods

Scattering methods are some of the most popular methods for determining molecular weight averages *M_w_*. The fundamentals of the light scattering phenomena were expounded by Lord Rayleigh in 1871. Light scattering (LS) is one of the few absolute methods that provide access to molecular weight and structure [72,73]. Light scattering is often used as a tandem technique together with separation using SEC [74]. As a standalone technique, it delivers the weight average *M_w_*, the corresponding z-average square radii *< r^2^_g_ >_z_*, and the second virial coefficient *A*_2_ of cellulose. Since it is an absolute method, it does not require calibration. One limitation of the LS method is that for a given concentration *c* (g∙mL^−1^), the scattered light signal is proportional to *c* × *M_w_*, such that molecules below a few thousand g∙mol^−1^ need relatively high concentrations in order to produce a detectable signal. Raising the concentration of, for example, a broad distributed sample, leads to problems, especially in the SEC portion of the process in which cellulose is analyzed. Dust will also scatter light and contribute to the intensity; therefore, dust has to be removed using ultra-fine filters or centrifugation prior to measurements. The source of radiation is, in most cases, visible light from a laser; therefore, using colored solvents (e.g., Cu- and Fe-containing complexes) is challenging and cellulose is often derivatized and dissolved in an organic solvent or colorless solvent instead. Thus far, DMAc/LiCl is the solvent of choice for an LS experiment coupled to an SEC. 

Measuring cellulosic samples with fluorescence activity (e.g., pulp with high lignin content) also poses some challenges. Due to the laser excitation, even small amounts of fluorophores will disrupt a measurement. There are two types of LS experiments: the batch mode and the chromatographic (online) measurement. In the batch mode (off-line), MALLS detector can be used as a standalone instrument to characterize an unfractionated polymer sample; the online use of an LS detector is described in Chapter 2.7. In this static light scattering experiment (SLS), a vertically polarized (laser) light is scattered by the macromolecules of a polymer in solution. The scattered light is detected by a photometer or a photodiode: either one photometer is used to encircle the sample in a horizontal plane or several photodiodes are placed around the measurement cell, which detects the scattered light from several positions and angles.

The ratio:

(17) $$R_{\text{θ}}=\frac{I_{scattered}}{I_{incident}}$$

depends on:

- the concentration of solution *c*;
- the specific refractive increment obtained at chemical equilibrium *dn*/*dc* or, more accurately, (*dn*/*dc*)µ;
- the molecular weight of dissolved particles (*M*);
- Scattering angle θ.

The scattering intensity is dependent on the angle and reflects the diminution of the light intensity by intra-particular interference. For light scattering experiments, the Zimm equation forms the basic for calculating the molecular weight:

(18) $$\frac{K^{*}c}{R(\text{θ},\;c)}=\frac{1}{M_{w}P(\text{θ})}+2A_{2}c$$

where K^*^ is the optical constant, *R*(θ, *c*) is the excess Rayleigh ratio of the solution as a function of scattering angle θ and concentration *c*, *M_w_* is the molar mass weight average, *P*(θ) is the angular dependence of the scattered light, *A*_2_ is the second virial coefficient, and *c* is the concentration of the solute.

The optical constant is described by the following equation:

(19) $$K^{*}=\frac{4*\pi^{2}{\left(dn/dc\right)}^{2}n_{0}^{2}}{N_{a}\lambda_{0}^{4}}$$

where *dn*/*dc* is the specific refractive increment at chemical equilibrium, *N_a_* is the Avogadro’s Number, λ_0 _is the wavelength of the incident light, and *n*_0_ is the solvent refractive index.

The measured data can be extrapolated to zero concentration and zero-degree scattering angle, which can be achieved for the batch mode by using the so-called Zimm-plot to derive three valuable quantities: the weight average *M_w_*, the second virial coefficient of osmotic pressure *A*_2_ (both with extrapolation to zero angle), and the radius of gyration (z-average) by extrapolating the concentration to zero. The specific refractive increment at chemical equilibrium must be measured separately by using a differential refractometer; practical details are provided in [28]. Insights into the theory and principles of LS can be found in the work of Wyatt and Flory [4,73].

LS alone, without prior separation, is used for polymer characterization in solution rather than for molecular weight determination. Zhou and his colleagues investigated cellulose dissolved in NaOH/urea aqueous solutions with LS and viscometry [75]. They calculated MHS parameters for this solvent and proposed its capability to dissolve cellulose between 3.0 × 10^4^ and 1.3 × 10^5^. Because this is also a nontoxic solvent, they emphasized its suitability for viscometry. Kamide and Saito dissolved acid-hydrolyzed cotton linters, regenerated from cuprammonium solution, in 6% LiOH and determined the molecular weight by LS and viscometry [76]. For structural clarification of cellulose in cuoxam, Seger and Burchard used MALLS [77]. Despite its deep blue color, they used cuoxam for LS experiments and were able to obtain molecular weight averages for cellulose. They measured at λ_0_ = 457.9 nm laser-wavelength and corrected the intensities for the absorption at this wavelength, which was determined by UV-Vis-spectroscopy.

Drechsler *et al*. demonstrated that static LS as a standalone device is useful for the molecular weight determination of cellulose dissolved in NMMO/H_2_O/diethylenetriamine (DETA) [78]. They compared the SLS results with SEC of the tricarbanilated cellulose (CTC). Higher DPs correlated with greater differences between SLS and SEC of CTC. The results from viscometry in cuen measurements were similar to the results from SLS in NMMO/H_2_O/DETA. The authors concluded that the degree of degradation in NMMO/ H_2_O/DETA is comparable to the degree of degradation in cuen.

**Figure 3:**
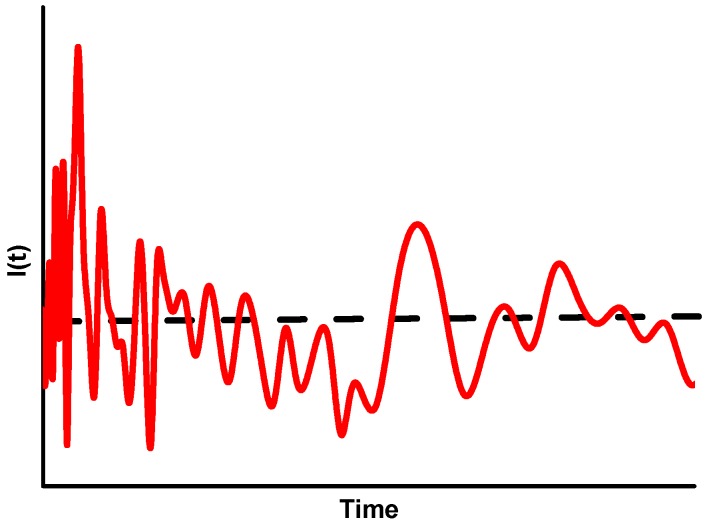
The fluctuation of scattered light over time (in the range of milliseconds). The dotted line represents the SLS-signal and the red line represents the DLS-signal.

SLS and dynamic light scattering are based on the same phenomena; in both cases, the scattering occurs at the same wavelength of the incident light. The only differences are the ways in which the experimental data are collected and processed. In DLS, the fluctuation of the scattered light is measured. The fluctuation is due to the Brownian motion of the scattering particles and occurs in extremely short time intervals. In SLS, the time-averaged intensity of the scattered light is measured (see Figure 3).

The measured fluctuation of the scattering reflects the motion of particles. As big particles move slowly and smaller ones move faster, fluctuation produces information about the size of the particles in motion. As with intrinsic viscosity measurements, DLS yields the hydrodynamic radii. In contrast to viscometry, DLS can also assess very small particles, down to 1 nm. For bigger particles, the acquisition time is extended. This limits the use of DLS in online measurements (e.g., SEC).

Quasi elastic light scattering (QELS) refers to photon correlation spectroscopy. It is a type of DLS that can be combined with a separation technique. In principal, a QELS photodiode can be replaced for a single angle in a MALLS detector (except for the 90° detector). Because the QELS photodiode is part of the same MALLS hardware, there is no need to measure the inter-detector delay. The primary drawback is the low sensitivity, which cannot always be compensated for by injecting higher analyte concentrations. To our knowledge, the only cellulose studies in which QELS was hyphenated with SEC were done by Yanagisawa *et al*. [79,80]. In those studies, the authors concluded that the conformation of cellulose and cellulose tricarbanilate (CTC) is the same in LiCl/DMAc as it is in LiCl/1,3-dimethyl-2-imidazolidinone (DMI).

### 2.6. Separating by Size—Size Exclusion Chromatography

Sometimes the plain molecular weight average bears the desired information. However, in order to access the distribution of the molecules, separation techniques are also required. Size exclusion chromatography (SEC) is currently the most highly recommended method in the field analysis of synthetic and biopolymers [72] and is by far the most common tool for determining the MWD of cellulose. Old methods like fractional precipitation are very tedious and suffer from poor reproducibility [81].

SEC (sometimes referred to as gel permeation chromatography or GPC) is a special type of column chromatography in which it is assumed that there are no enthalpic interactions between the analyte and the stationary phase. Molecules are only separated according to their hydrodynamic volume. In contrast to other chromatography techniques, large molecules (in terms of hydrodynamic volume) are eluted first, whereas small molecules will enter the pores of the solid porous phase and elute later. The hydrodynamic radius is the space required by a molecule in solution and it changes based on the solvent used. The interaction between molecule and solvent, conformation of the molecule, and branching all influence the hydrodynamic radius to a great extent. It is possible that molecules of different origin with diverse molecular weights could have the same hydrodynamic radius. In addition, different molecules having the same molecular weight can have a different hydrodynamic radius. The hydrodynamic radius influences retention time; therefore, molecules with different molecular weights could have similar retention times. This fact can lead to a miscalculation of molecular weights if they are determined only by retention times. To summarize, SEC results in a separation of the molecules according to their hydrodynamic volume. The molecular weight, however, is more frequently of interest than the molecular size. Therefore, an SEC system must be calibrated unless an absolute detection system is applied. Almin *et al*. [82] conducted one of the rare studies on direct calibration of SEC with cellulose standards. They used preparative SEC (GPC) to fractionate cellulose dissolved in cadoxen. After fractionation, they dissolved the fractions again in cadoxen, measured the molecular weight offline by means of viscometry, and calibrated SEC agarose gel columns. With the developed method, they were able to correct for the band-broadening that occurs in SEC, as the gel material they employed had a notably poor resolution (especially at high molecular weight), and it is doubtful that there were no interactions between the stationary phase and analyte, as proposed by the authors.

Both an overview paper by Kostanski *et al*. and a book by Striegel *et al*. provide a summary of possibilities for calibrating SEC [83,84]. Detection systems used in SEC can be divided into concentration detectors and molecular weight sensitive detectors. For quantitative measurements, at least one concentration detector must to be used, and that will most commonly be a detector measuring the refractive index (RI). The response of an RI detector is normally independent of molecular weight, but it is important to remember that with very low molecular weight levels, this response can change. For some polymers, it is known how the refractive increment changes in the oligomeric region; however, for cellulose, these data are not yet available (see Chapter 2.8). In most cases, detectors that measure UV or IR absorption are not of interest in cellulose SEC experiments. When measuring functional groups within the cellulose molecule (carbonyl and carboxyl groups), a fluorescence detector is applied. After derivatization, such groups can be measured in an SEC-MALLS setup simultaneously with the MWD [85]. In addition to the LS coupling discussed below, viscosity detectors such as differential viscometers and single- or dual-capillary viscometers also serve as molecular weight sensitive detectors, provided that the Mark-Houwink constant of the analyzed polymer is known. However, a viscosity detector shows greater sensitivity to high molecular weight solutions, which enables the oligomeric portion to be distinguished. In principle, the concept of a universal calibration could solve this problem; however, this calibration is also not without its problems, chief among them the fact that an exact amount of polymer must be injected in order to calculate peak integrals exactly. Finally, a universal calibration is only valid if the separation process is based solely on entropic effects. Thus, if there are other interactions between the polymer and the stationary phase, universal calibration is not applicable. Strlic investigated the enthalpic interaction on cellulose during SEC using cross-linked styrene–divinylbenzene gel columns [20]. Enthalpic interactions can make a universal or a relative calibration invalid. In such a case, LS detection can offer an alternative, provided that enthalpic effects do not lead to local dispersity. Unfortunately, an LS detector is not always available. Due to the lack of commercially available cellulose standards with narrow distribution, an alternative is calibration with the linear polysaccharide pullulan (maltotriose units which are linked α-1,6) [86,87,88]. SEC without absolute measurements can therefore give rise to a systematic error that is higher than a random error [28].

The concept of a universal calibration uses the hydrodynamic volume as a calibration parameter according to the Einstein viscosity law. It was first proposed by Benoit *et al*. [89]:

(20) [η]=K(V/M)

where [η] is the limiting viscosity, *V* the hydrodynamic volume of the particle, *M* the molecular weight, and *K* is a constant. The Einstein viscosity law shows that the product [η] × *M* is a direct measure of the hydrodynamic volume of the molecule, so that *log* [η] × *M* can be used instead of *log M* in the calibration of SEC systems. Using polystyrene to create a universal calibration for cellulose (and derivatives) could lead to errors [90] and is more accurately described as pseudo-universal. However, if an absolute detector is not available or only relative comparison of samples are needed, universal calibration offers a satisfactory alternative [91].

### 2.7. A Powerful Combination: SEC-MALLS

Molecular weight sensitive detectors are essential for providing molecular weight information, especially when standards are missing, as is the case for cellulose or when complex polymers have to be analyzed. Advanced systems utilize the MALLS-RI detector setup for obtaining the molecular weight and the MWD in cellulose analytics without additional calibration standards [74]. Multi-angle LS is by far the most common LS detector for the measurement of molecular weight distributions and molecular weight averages. The utilization of LALS or even right angle light scattering (RALS) is rather rare in the analysis of non-derivatized cellulose [26,92,93]. In an SEC-MALLS setup, the RI-detector is required to determine the concentration of the analyte. It is a universal detector, which means that it recognizes every difference between the pure solvent and the sample solution. Through the specific refractive index *dn*/*dc*, it is possible to calculate the concentration of the sample.

As the full Zimm plot can only be constructed from batch mode experiments (see above), a reduced plot must be used for the chromatographic approach. The Debye plot, which has the same coordinates as the Zimm plot, uses only one concentration. For each slice of a peak, which can be considered a uniform fraction, a Debye Plot can be constructed. There are three possibilities for building such a Debye plot: the Zimm formalism (which is largely applied in cellulose analytics as it is best for mid-sized polymers (rms 10–100 nm), the Debye formalism for small polymers (rms < 50 nm); and the Berry formalism for large polymers (rms 100–200 nm).

For the characterization of cellulose, SEC standards continue to be a nagging concern [94,95,96]. As noted above, LS detection can overcome this problem as long as *dn*/*dc* is known. Again, *dn*/*dc* is a constant for polymers but can vary for low molecular weight cellulose (with hemicelluloses present) or oligomers. Another drawback of LS analysis of low molecular weight cellulose (below ~ 3000) is the fact that high concentrations are needed to get signals from such short molecules. However, in the analysis of paper aging, pulping, bleaching, enzymatic treatment, and the viscose process, SEC with DMAc/LiCl as a solvent and eluent is the technique of choice [87,97,98,99,100,101] and has been reviewed extensively [28,87,102]. DMAc/LiCl was introduced for use in dissolving polyamides and chitin in 1972; almost concomitantly, both McCormick and Turbak patented a dissolution process for cellulose in this solvent system. In the 1980s, Ekmanis first proposed this system for the SEC analysis of cellulose [18]. Solvents commonly used for viscometry are usually incompatible with SEC columns. Heywood *et al*. reported problems when using cadoxen as an SEC eluent with Fractogel TSK (Merck) [103], but Schwald *et al*. used cadoxen successfully in SEC experiments using PSS Suprema (PSS) column packing [104]. Iron tartrate as a cellulose solvent was also reported as an eluent [105]. However, DMAc/LiCl has clear advantages linked to direct dissolution and stability in this system and for oxidized samples. Therefore, this chapter will focus on SEC-MALLS coupling with DMAc/LiCl as the polymer solvent. 

Before cellulose is dissolved in DMAc/LiCl, it has to be activated. There are several protocols and papers that deal with this activation step. However, cellulose is often activated after a solvent exchange with hot (above 70 °C) DMAc. Potthast *et al*. showed that cellulose undergoes depolymerization during this step [106]. The actual relative proportions of LiCl and cellulose are also critical to the dissolution process; in general, aggregation of the polymer occurs (and was reviewed by Sjöholm) [107]. Potthast *et al.* found the limiting concentration of LiCl in DMAc to be 8.46%; therefore, earlier data has to be re-evaluated, taking the water content of the system into account [21].

Bikova *et al.* discussed the problems of an SEC analysis of cellulose in their review. Although DMAc/LiCl is reported to be a non-degrading solvent and is commonly used, they noted six general problems with using this solvent. They proposed a pseudo-size exclusion with the DMAc/LiCl system because of the polyelectrolyte behavior of cellulose in DMAc/LiCl, over-salting, and salt sensitivity. The six general problems noted by Bikova *et al*. were:

- A lack of studies on the elution behavior of cellulose in which the degree of oxidation and the content of hemicelluloses and lignin are considered;
- Optimization of the salt concentration in order to eliminate all distorting interactions such as macromolecule interactions and polyelectrolytes;
- Re-examination of the SEC mechanism of the calibrants pullulan and polystyrene;
- Correct Mark-Houwink constants for cellulose in DMAc/x*LiCl/y*H_2_O at given temperatures;
- Experimental proof of the validity of the universal calibration;
- Replacing DMAc/LiCl as an eluent [79,108].

Despite these challenges, using DMAc/LiCl as a dissolution solvent and as an eluent has major advantages compared to other solvent types.

### 2.8. The Gloomy Part—Capturing the Low Molecular Weight Region

Low molecular weight cellulose and cello-oligomers are not given much consideration, despite their great potential in current and future technologies. Such molecules can be seen as degradation products in the pulp and paper industry or as intermediates in a total hydrolysis of the polymer. Strength properties and the ability to form films or fibers are largely influenced by higher molecular weights; hence, such properties are closely related to molecular weight distribution. As a consequence, low molecular cellulose and the oligomeric region of cellulose have not received much attention in cellulose analytics or in polymer analytics in general.

An exact definition—a DP value at which the molecules are referred to as oligomers or polymers—remains elusive. A common distinctive feature is water solubility, which for cellulose means that the water-soluble saccharides (DP ≤ 8) are referred to as cello-oligosaccharides and the insoluble saccharides with higher DPs are referred to as polysaccharides. Since solubility in water is also a question of concentration and other DP-dependent properties do not change as rapidly, this strict demarcation is neither especially precise nor applicable. Thus, the authors refer to cello-oligomers as molecules with a DP up to 30–35.

The separation and quantitation of (cello-)oligomers is a great challenge. In order to obtain an accurate view of the entire molecule, it is necessary to analyze both the oligomeric and the polymeric levels. Several studies have addressed the analysis and preparation of cello-oligomers. For a detailed overview, see [109]. To our knowledge, no research has been conducted to determine an accurate quantification of the oligomeric portion of the cellulose molecule. Simms presents a high performance liquid chromatography (HPLC) method using a β-cyclodextrin-bonded stationary phase [110]. He investigated neutral oligosaccharides and showed a separation of cello-oligomers up to a DP of 8. Churms uses a particulate polyacrylamide (PAA) for a preparative SEC of cello-oligomers [111]. These gels are the most frequently used stationary phases in these efforts and are advantageous in terms of selectivity, resolution, low bond broadening, and linearity between the logarithm of the distribution coefficients and the degree of polymerization [109]. The main drawback of using PAA gels is their lack of resistance to high pressures, resulting in long separation times of 24 h or more [111,112]. Thus, these gels are used on the preparative side and not for analytics. Isogai *et al*. [113] analyzed the DP of cellouronic acids, prepared from alkali and ball-milled treatment of cellulose after TEMPO oxidation, dissolved in 0.1 M NaCl on polyhydroxymethacrylate gels. Native cello-oligomers were not reported as analyzed. In general, semi-analytics and preparation of cello-oligosaccharides below DP 8 could be achieved by different methods, whether SEC, anion- or cationic exchange chromatography, sugar boronate affinity chromatography, normal phase HPLC, or hydrophilic interaction chromatography [112,114,115,116,117,118,119].

In solution, oligomers are known to exhibit different behavior than their corresponding polymers. Striegel showed that polyethylene oligomers in trichlorobenzene at elevated temperatures and functionalized and non-functionalized polystyrene in DMAc/LiCl 0.5% (35 °C) have negative viscosities [120]. Glucose in DMAc/LiCl 0.5% was shown in the same study to have a positive viscosity. There are numerous examples showing that optical properties are a function of molecular weight. For example, oligomers of methacrylate yielded different refractive indices between the monomer and the oligomer [121].

For cello-oligomers, such studies are still very rare, most likely because uniform standards are effectively unavailable and the utilization of such molecules is not especially pronounced. However, such standard compounds would offer the opportunity for new experiments and methods. In addition, the separation and quantification of low molecular weight cellulose is an important issue when utilizing such degraded polymers.

Morris and Striegel investigated the conformational entropy of (cello-)oligosaccharides using SEC-viscometry [122]. They demonstrated that even in this DP range (DP 2–6), the viscometer produces a satisfactory response. In their conclusion, they suggested that a viscometer’s response is independent of the molecular weight.

LS techniques are often said to reach their limits by measuring polymers of molecular weight much below 10,000 g∙mol^−1^, largely because the scattered light signal is proportional to *c* × *M_w_*. However, there is one claim from a MALLS instruments manufacturer that a uniform polystyrene sample with a molecular weight of 580 g∙mol^−1^ can be accurately measured by means of SEC-MALLS [123]. In the literature, the proof of the suitability using light scattering for small molecules is minimal. Xie produced one paper with SEC-MALLS down to 2500 g∙mol^−1^, showing that SEC-MALLS of poly(diisopropyltrimethylene-1,1-dicarboxylate) with chloroform as an eluent is possible down to the oligomeric range [124]. The results were compared with data obtained by matrix-assisted laser desorption ionization-time of flight mass spectrometry (MALDI-TOF MS), nuclear magnetic resonance (NMR), and vapor pressure osmometry. All values were in agreement; however, for cellulose in DMAc/LiCl, MALLS detection is known to have its limit below approx. 3000 g∙mol^−1^.

### 2.9. Evaluation of MWD Curves

**Figure 4:**
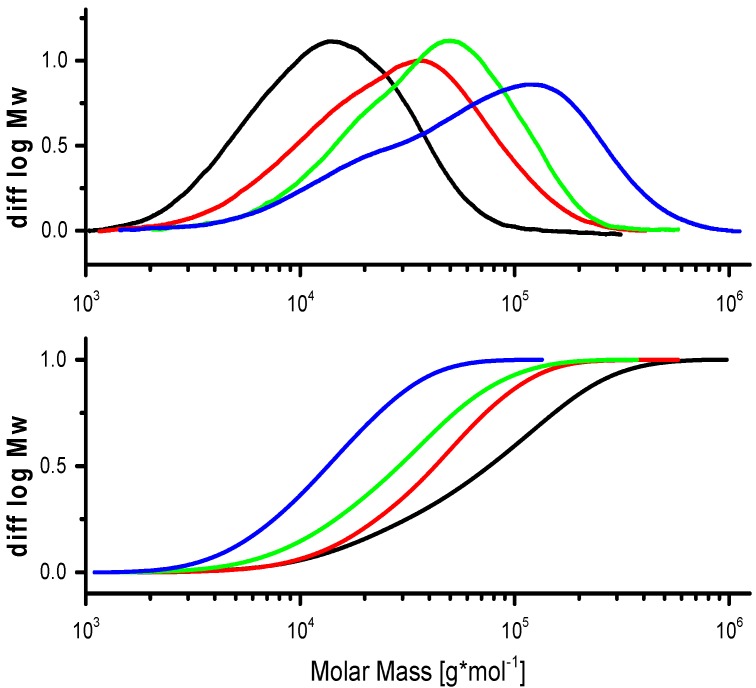
Differential (top) *vs.* cumulative distribution of molar mass (cellulose degradation kinetics).

Once the MWD is measured, it yields a definite surplus of information compared to molecular weight averages. Explaining and comparing MWD is not as easy as simply looking at sum parameters. The ability to extract important information from distributions is often also related to experience and empirical knowledge. As pointed out in Section 1.2, two plots are common in the polymer community for displaying MWD: the cumulative and the differential distribution are used to describe the MWD graphically (Figure 4). The differential distribution is mostly used in polymer analytics. It gives a quick overview of the MWD symmetry, multimodal distributions, the molar mass minimum resp. maximum, and the molar mass of the most abundant fractions. With the cumulative plot, it is easy to determine the weight fraction above or below a certain molar mass limit. However, the differential plot is preferred in polymer analytics simply because it yields more information. The differential plot in Figure 4 demonstrates the bimodality of the sample (blue line), whereas with a cumulative plot, such information gets lost.

**Figure 5:**
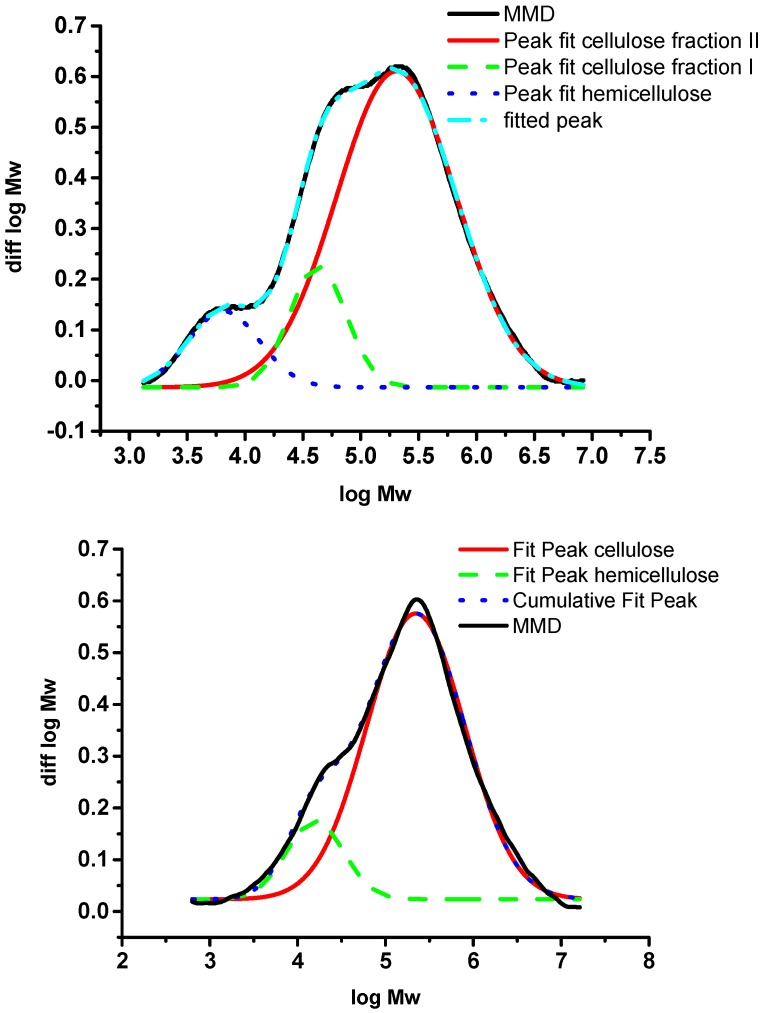
MWD of a hardwood pulp (**top**) and a softwood pulp (**bottom**) with multiple-peak fits of the cellulose and hemicellulose fraction [34].

In the following, we will discuss the distribution and multiple-peak fitting of two different celluloses to show the importance of looking at the MWD and, if necessary, to perform multiple peak fitting in overlapping peaks. Example one is a dissolving pulp cellulose made from hardwood which has been prepared under acid conditions by the sulfite process (Figure 5). The pH during pulping is between 2–3, at temperatures above 100 °C. After a totally chlorine free (TCF) bleaching sequence, a MWD as given in Figure 5 is obtained. The cellulose shows a trimodal distribution if the measurement settings allow for a good resolution. The cellulose part between log MW 4.5 and 6.5 shows two slightly separated peaks that originated from the acid conditions, which, due to hydrolysis, led to two different populations of the bulk cellulose. The smaller peak between log MW 3 and 4 corresponds to the remaining hemicellulose and a fraction of degraded cellulose. The hemicellulose peak in the low molar mass area is better separated from the cellulose fraction; in some cases, such as eucalyptus kraft pulp cellulose, it can be almost fully separated. This behavior is very pronounced for hardwood pulps and applies to paper and dissolving pulp celluloses. The reason for the good separation here is the different dissolution structure of xylan, the predominant hardwood hemicellulose, in the solvent. After multiple-peak fitting of the RI signal, the hemicellulose peaks account for approximately 8% of the total peak area (blue line in Figure 5, top). This is in agreement with data from methanolysis, which show about 3.2% total xylose content. The remaining difference can be explained be degraded cellulose fragments (cello-oligosaccharides) which cannot be distinguished from cellulose in a total hydrolysis or methanolysis experiment. In cases where there are similar amounts of glucomannan, the structural similarity between glucomannan and smaller cellulose fragments leads to a less effective separation in SEC. Here, a multiple-peak fitting can be helpful to approximate mass fractions of hemicelluloses. An example for a softwood dissolving pulp is given in Figure 5 (bottom).

## 3. Conclusions and Outlook 

This review has outlined methods for the molecular weight determination of cellulose and has taken older methods and historical approaches into account. Some of the older methods will remain more or less of historical interest or are only used now in very specific cases such as obtaining detailed information on the structure in solution. However, the main limitation of all methods is the solubility of the polymer. New solvents, like the ionic liquids that were introduced recently in cellulose chemistry, are also of interest in the analytical field but have not yet reached the level of application in SEC. There is clearly a lack of analytical methods for low-molecular mass cellulose. There is also a lack of knowledge on the viscosity of solutions containing high amounts of degraded cellulose fragments. The Mark-Houwink parameters are known to be susceptible to change with small molecules, leading to errors in viscosity measurements, and it is not yet clear when these parameters begin to change. The same is true for the refractive increment, which is only a constant for polymers. Comparative studies of different methods for determining molecular weight are still rare, mainly because an evaluation of methods in this field demands dramatic complexity involving a significant number of different parameters.

The neglected oligomeric part of (hemi-)cellulose is an issue for several reasons, including the fact that side streams, as found in pulping and paper processing, can be a valuable source for small (hemi)cellulose fragments. Before processing such small molecules, however, a proper analytic is a minimum requirement. The influence of oligomeric compounds on a polymer product and its properties is still not clearly understood. Therefore, new methods are needed which will allow the clarification of the low molecular weight portion of the cellulose molecule. In the future, only powerful solvents compatible with advanced analytical systems will enable a real step forward. For the solvents, this requires a rather low viscosity, no interference with absolute detection systems (no fluorescence, no absorption, *etc*.), and chemical compatibility with all elements of system hardware. The currently used salt-containing solvents cause manifold problems in that respect. The application of alternative separation methods like field flow fractionation (FFF) techniques would be beneficial in avoiding expensive sets of separation columns but are not yet compatible with cellulose solvents.
